# Two distinct conformers of PrP^D^ type 1 of sporadic Creutzfeldt–Jakob disease with codon 129VV genotype faithfully propagate in vivo

**DOI:** 10.1186/s40478-021-01132-7

**Published:** 2021-03-25

**Authors:** Ignazio Cali, Juan Carlos Espinosa, Satish K. Nemani, Alba Marin-Moreno, Manuel V. Camacho, Rabail Aslam, Tetsuyuki Kitamoto, Brian S. Appleby, Juan Maria Torres, Pierluigi Gambetti

**Affiliations:** 1 Department of Pathology, School of Medicine, Case Western Reserve University, Cleveland, OH USA; 2 Department of Neurology, School of Medicine, Case Western Reserve University, Cleveland, OH USA; 3 Department of Psychiatry, School of Medicine, Case Western Reserve University, Cleveland, OH USA; 4 National Prion Disease Pathology Surveillance Center, School of Medicine, Case Western Reserve University, Cleveland, OH USA; 5 Centro de Investigación en Sanidad Animal, CISA-INIA, Madrid, Spain; 6 Centre for Prions and Protein Folding Diseases, Brain and Aging Research Building, University of Alberta, Edmonton, Canada; 7 Department of Neurological Science, Tohoku University Graduate School of Medicine, Sendai, Japan

**Keywords:** Prion protein, sCJDVV1, Prion strain, Histotype, Transmission properties, Lesion profile, Plaques

## Abstract

Current classifications of sporadic Creutzfeldt–Jakob disease (sCJD) identify five subtypes associated with different disease phenotypes. Most of these histopathological phenotypes (histotypes) co-distribute with distinct pairings of methionine (M)/valine (V) genotypes at codon 129 of the prion protein (PrP) gene and the type (1 or 2) of the disease-associated PrP (PrP^D^). Types 1 and 2 are defined by the molecular mass (~ 21 kDa and ~ 19 kDa, respectively) of the unglycosylated isoform of the proteinase K-resistant PrP^D^ (resPrP^D^). We recently reported that the sCJDVV1 subtype (129VV homozygosity paired with PrP^D^ type 1, T1) shows an electrophoretic profile where the resPrP^D^ unglycosylated isoform is characterized by either one of two single bands of ~ 20 kDa (T1^20^) and ~ 21 kDa (T1^21^), or a doublet of ~ 21–20 kDa (T1^21−20^). We also showed that T1^20^ and T1^21^ in sCJDVV have different conformational features but are associated with indistinguishable histotypes. The presence of three distinct molecular profiles of T1 is unique and raises the issue as to whether T1^20^ and T1^21^ represent distinct prion strains. To answer this question, brain homogenates from sCJDVV cases harboring each of the three resPrP^D^ profiles, were inoculated to transgenic (Tg) mice expressing the human PrP-129M or PrP-129V genotypes. We found that T1^20^ and T1^21^ were faithfully replicated in Tg129V mice. Electrophoretic profile and incubation period of mice challenged with T1^21−20^ resembled those of mice inoculated with T1^21^ and T1^20^, respectively. As in sCJDVV1, Tg129V mice challenged with T1^21^ and T1^20^ generated virtually undistinguishable histotypes. In Tg129M mice, T1^21^ was not replicated while T1^20^ and T1^21−20^ generated a ~ 21–20  kDa doublet after lengthier incubation periods. On second passage, Tg129M mice incubation periods and regional PrP accumulation significantly differed in T1^20^ and T1^21−20^ challenged mice. Combined, these data indicate that T1^21^ and T1^20^ resPrP^D^ represent distinct human prion strains associated with partially overlapping histotypes.

**Supplementary Information:**

The online version contains supplementary material available at 10.1186/s40478-021-01132-7.

## Introduction

For several years sporadic Creutzfeldt–Jakob disease (sCJD) has been grouped into five distinct subtypes, denoted as sCJDMM(MV)1, -MM2, -MV2, -VV1 and -VV2 [18, 38]. This grouping is based on the combination of two major molecular determinants of the disease phenotype: the methionine (M)/valine (V) polymorphic genotype at codon 129 of the prion protein (PrP), which dictates the MM, MV and VV 129 genotypes, and the type 1 or 2 of the disease-associated PrP (PrP^D^) [7, 8, 31, 38]. PrP^D^ types 1 (T1) and 2 (T2) are distinguished by their respective ~ 21 kDa and ~ 19 kDa electrophoretic mobilities following treatment with proteinase K (PK), which are commonly monitored (for practical reasons) with the unglycosylated isoform (lower band) of the PK-resistant PrP^D^ (resPrP^D^) [17]. The distinct mobility reflects the different sizes of the PK-resistant region and, therefore, the distinct conformations of the T1 and T2 PrP^D^ isoforms [31, 36, 40].

In this study, which is part of a body of research on sCJD subtypes, we focus on sCJDVV1, the least investigated subtype, especially with regard to the characteristics of its PrP^D^ [11, 37].

Sporadic CJDVV1 is also the rarest of the five subtypes, accounting for 2–3% of sCJD [11, 39, 46]; it presents at a younger age on average, with clinical onset often in the 3th or 4th decade of life, and has a relatively long course that often exceeds one year [11, 18, 41]. Phenotypically, sCJDVV1 is easily distinguishable from the other sCJD subtypes by the type and distribution of the histological lesions (histotype), which include severe spongiform degeneration (SD) with medium size vacuoles throughout the cerebral cortex, presence of ballooned neurons and widespread but light PrP deposition [11]. The electrophoretic profile of sCJDVV1 resPrP^D^ T1 is complex, as shown by the heterogeneity of the unglycosylated isoform. We recently identified three alternative electrophoretic profiles or variants of T1: the T1^20^ and T1^21^ variants, where the two resPrP^D^ fragments of ~ 21 and ~ 20 kDa occur separately, and the T1^21−20^ variant where the two resPrP^D^ fragments coexist in different ratios [11]. We also observed that T1^21^ and T1^20^ have distinct conformational characteristics suggesting that they represent distinct strains. Nonetheless, the histotypes associated with the T1^20^, T1^21^ and T1^21−20^ variants are similar violating the tenet that distinct prion strains are associated with distinct phenotypes [6, 17, 42].

To further investigate this issue, transgenic (Tg) mice expressing normal or cellular human PrP (PrP^C^) with the codon 129 residue V (Tg129V) or M (Tg129M), were inoculated with sCJDVV1 brain isolates containing T1^20^, T1^21^ or T1^21−20^* (the last isolate was obtained from a sCJDVV1-2 case harboring tiny amounts of T2, denoted by asterisk). Brain extracts from sCJDVV2, a different sCJD subtype that harbors resPrP^D^ T2 (with a ~ 19 kDa unglycosylated fragment), were inoculated as controls. Both T1^20^ and T1^21^ were faithfully replicated in Tg129V mice with T1^21^ showing a longer incubation period, whereas T1^21−20^* was reproduced as T1^21^. Replication was longer and less faithful in the Tg129M mice: the ~ 21–20 kDa resPrP^D^ doublet was generated following inoculations with T1^20^, and T1^21^ was not transmitted. The histotype in T1^20^ and T1^21−20^*-inoculated Tg129M mice was characterized by the overlapping lesion profiles and the lack, in T1^21−20^*-inoculated mice only, of PrP deposits in cerebral cortex and cerebellum. Second passage in Tg129M mice recapitulated the results of the first passage except for the significantly shorter and different incubation periods of T1^20^ and T1^21−20^*-infected mice.

The transmission in Tg129V mice of both T1^21^ and T1^20^ with the accurate replication of their electrophoretic profiles, along with the lack of replication of T1^21^ only following serial transmissions in Tg129M mice suggest that T1^21^ and T1^20^ are distinct prion strains even though they are associated with similar histotypes in sCJDVV1.

## Materials and methods

### sCJDVV case-patients

Four cases of sCJDVV1, one case of sCJDVV1-2 and one case of sCJDVV2 (cases 2–5, 16 and 6, respectively, of Table S2 of Cali et al. [11]; Additional file 5: Table S1 of the present study) were used as inocula for the transmission study. Inocula were generated from the frontal cortex (sCJDVV1, N = 3; sCJDVV2, N = 1), parietal cortex (sCJDVV1, N = 1), occipital cortex (sCJDVV1-2, N = 1) and putamen (sCJDVV2, N = 1) (Additional file 5: Table S1). All samples were obtained from the National Prion Disease Pathology Surveillance Center (NPDPSC) in Cleveland, USA.

### Features of resPrP^D^ of the inocula

The inocula containing resPrP^D^ T1^20^ were obtained from three cases of sCJDVV1 while inocula harboring T1^21^ and T2 were each isolated from one case of sCJDVV1 and sCJDVV2, respectively; T2 was used as control. T1^21−20^* corresponds to T1 variant with a ~ 21–20  kDa doublet co-existing with T2 (the latter accounting for ~ 5% of the total resPrP^D^) harvested from a case of sCJDVV1-2 that had histotype mostly consistent with sCJDVV1 (Additional file 5: Table S1). Immunoblotting characterization of the inocula confirmed the previously established electrophoretic profiles of T1^20^ and T1^21^ resPrP^D^ variants and excluded the presence of T2 (Additional file 1: Figure S1). The consistent predominance of the ~ 21 kDa component in the T1^21−20^* variant was also confirmed. Of note, the small T2 component of T1^21−20^* was detected with the T2-specific Ab Tohoku-2 (data not shown), but not with the type generic 3F4 (Additional file 1: Figure S1). T2 in sCJDVV2 was harvested from the frontal cortex and putamen, which were used as separate inocula.

### Transgenic mice

Two Tg mouse lines, the Tg362 and Tg340, were used [34, 35]. They express the human PrP^C^-129V (Tg362) and PrP^C^-129M (Tg340) at ~ eightfold and ~ fourfold normal human brain levels, respectively, and are hereafter identified as Tg129V and Tg129M.

### Intracerebral inoculations

Twenty microliters of ten percent (wt/vol) brain homogenates (BH) in 5% glucose generated from the sCJDVV cases were inoculated intracerebrally according to previously described procedures [35]. A total of 99 Tg mice were inoculated in this study. Brain homogenates from Tg129M mice challenged with each of the three T1 variants were used for a second passage in the same mouse line.

### Histology, immunohistochemistry, lesion profiles, and morphometric analysis

Histological and immunohistochemical examinations were carried out on four brain levels at approximately bregma 0.5 mm,  − 1.7 mm, − 3.8 mm and − 6.0 mm, as previously described [10]. Paraffin sections were stained with hematoxylin and eosin (H.E.) or probed with the Ab 3F4 [22, 47] to human PrP (residues 106–110) at 1:1,000 and 1:400 dilutions as previously described [10]. Lesion profiles were performed using semi-quantitative evaluation for severity of SD, which was rated on a 0–3 scale on H.E.-stained sections (0 = not detectable; 1 = mild, 2 = moderate, and 3 = severe) [14]. Each point of the lesion profiles and bar graphs in Figs. 2 and Additional file 3: S3 were expressed as mean ± standard error of the mean (SEM). The eight brain regions examined included the cerebral cortex, hippocampus, basal ganglia, thalamus, hypothalamus, superior and inferior brainstem, and cerebellum. The semi-quantitative assessment of gliosis severity and neuronal loss in the cerebellum was rated on a 0–3 scale as noted above. Morphometric analysis to assess vacuole-size was carried out on the cerebral cortex at the level of bregma − 1.7 mm, and measured by the software Image-Pro Plus (Media Cybernetics, Inc.) [23].

### Preparation of brain homogenates, PK digestion and Western blot analysis

Ten percent (wt/vol) BH of human cases were prepared using 1X LB100 (100 mM NaCl, 0.5% Nonidet P-40, 0.5% sodium deoxycholate, 10 mM EDTA, 100 mM Tris–HCl, pH 8.0), and supernatants (S1) were collected following centrifugation at 1000 × g for 5 min (min) at 4 °C. For the mouse brains, 10% BH prepared in 5% glucose was mixed with an equal volume of 2X LB100 (pH 8.0) and centrifuged at 1000 x g for 5 min. Human and mouse S1 were subjected to enzymatic digestion with 10 Units/ml (U/ml) PK (Sigma Aldrich), which was used at 48 U/mg PK specific activity (1 U/ml is equal to 20.8 μg/ml PK) at 37 °C for 1 h (h). Enzymatic reaction was stopped by the addition of 2 mM phenylmethylsulfonyl fluoride (PMSF) prior to the dilution of each sample with an equal volume of 2 × Laemmli buffer (6% SDS, 20% glycerol, 4 mM EDTA, 5% β –mercaptoethanol, 125 mM Tris–HCl, pH 6.8) and then denaturation at 100 °C for 10 min.

Proteins from human S1 were separated on 15% Tris–HCl SDS–polyacrylamide long gels (W x L: 20 cm × 20 cm) (Bio-Rad PROTEAN® II xi cell system) as originally described [9]. Proteins from the mouse S1 were separated on 15% Criterion™ Tris–HCl Precast Gels (W × L: 13.3 cm × 8.7 cm)[9] (Bio-Rad Laboratories, Hercules, CA, USA). Proteins were blotted onto the Immobilon-FL PVDF membrane (8.7 cm-long gels) or Immobilon-P PVDF membrane (20 cm-long gels) (EMD-Millipore, Billerica, MA, USA), blocked with a blocking buffer and probed with Ab 3F4 (1:10,000), 1E4 (1:500) and Tohoku-2 (1:10,000). The Ab Tohoku-2 was kindly provided by Dr. Tetsuyuki Kitamoto [28]. Membranes were developed by (1) the enhanced chemiluminesce reaction using ECL and ECL plus reagents, and signal was captured on MR and XAR films (20 cm-long gels), or (2) by the Odyssey infrared imaging system (LICOR Biosciences) (8.7 cm-long gels) as described by the manufacturer.

## Results

### Transmission features and characterization of resPrP^D^ variants in Tg mice

**Fig. 1:**
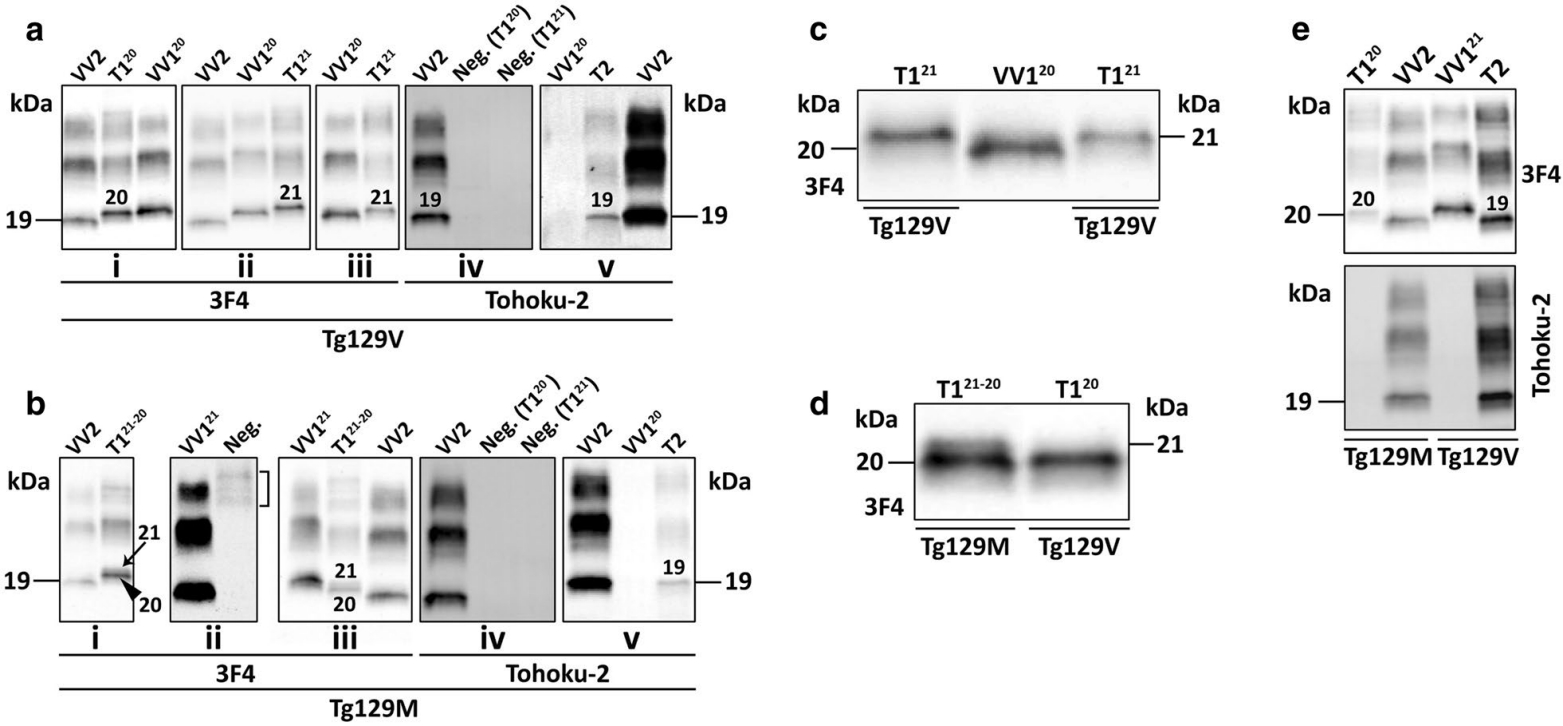
Characterization of mouse brain resPrP^D^. T1 and T2 and their superscripts atop of each blot refer to the mouse resPrP^D^ type and variant; VV1^20^, VV1^21^ and VV2 refer to resPrP^D^ obtained from sCJDVV cases. **a**, **i-ii**: The unglycosylated isoform of the mouse resPrP^D^ migrated to either ~ 20 kDa (**i**, “20”) or ~ 21 kDa (**ii**, “21”) accurately replicating the resPrP^D^ of VV1^20^ and VV1^21^, respectively; **iii** and **v**: VV1^21−20^* inoculum was reproduced as T1^21^ (**iii**, “21”) with the additional presence of a Tohoku-2-immunoreactive ~ 19 kDa band (**v**, “19”); **iv**: Tohoku-2 showing a negative (Neg.) immunoreactivity for T1^20^ and T1^21^ that were detected by 3F4 in **i** and **ii**, respectively. **b**, **i** and **iii**: Mice challenged with VV1^20^ (**i**) and VV1^21−20^* (**iii**) generated resPrP^D^ T1^21−20^ with a predominant ~ 20 kDa fragment (arrowhead, **i**) over a ~ 21 kDa band (arrow, **i**). **ii**: Mice challenged with VV1^21^ were all negative; bracket: mouse immunoglobulins. **iv** and **v**: Tohoku-2 immunoreacted with T2 in mice challenged with VV1^21−20^* (**v**) but not in those inoculated with VV1^20^ or VV1^21^ (**iv**). **c**-**d**: Magnification of unglycosylated resPrP^D^ fragments. **C**: Mouse resPrP^D^ migrated to ~ 21 kDa in Tg129V inoculated with VV1^21^ (lane 1) or VV1^21−20^* (lane 3). **d**: A small fragment of ~ 21 kDa migrating above a prominent one of ~ 20 kDa (lane 1), or a single band of ~ 20 kDa (lane 2), was detected in Tg129M or Tg129V inoculated with VV1^20^. **e**: Mice challenged with VV2. Top panel, Ab 3F4: Mouse resPrP^D^ migrated to either ~ 19 kDa (“19”) in Tg129V or ~ 20 kDa (“20”) in Tg129M. Bottom panel, Ab Tohoku-2: The mouse resPrP^D^ of  ~ 19 kDa, but not the ~ 20 kDa band, was detected by Tohoku-2

In Tg129V mice, all three T1 variants and T2 transmitted with 100% attack rate. Incubation periods or days post inoculation (dpi) varied; it was the longest for T1^21^ (425 ± 66 dpi) and the shortest for T1^21−20*^ (315 ± 66 dpi) even though the difference did not rich statistical significance (Tables 1 and Additional file 5: S2). T2 transmitted in 215 ± 18 dpi, which was significantly different (*P* < 0.0001) from the incubations of all T1 variants combined. T1^20^ and T1^21^ electrophoretic mobility and Ab immunoreactivity were indistinguishable from those of the respective inocula (Fig. 1a, c, d). By contrast, the T1^21−20^* inoculum (with predominance of the T1^21^ component and presence of ~ 5% T2) was reproduced only as T1^21^, with the addition of a weak band of ~ 19 kDa that was detectable with the T2-specific Ab Tohoku-2 but not with the type generic 3F4, mirroring the T2 of the inoculum (Table 1 and Fig. 1a). T2 was faithfully replicated as the typical ~ 19 kDa resPrP^D^ T2 (Fig. 1e). Ancillary transmission studies with hemizygous Tg129V mice challenged with T1^20^ and T2 isolates led to results similar to those obtained with homozygous mice with the exception of longer incubation periods (data not shown).

**Table 1:** Transmission features of sCJDVV resPrP^D^ to Tg129V and Tg129M mice

**Tg129V** (1st pass.)				
Inoculum	VV1^20^	VV1^21^	VV1^21−20*^	VV2
Attack rate (%)	100	100	100	100
Incubation (dpi)	351 ± 16	425 ± 66	315 ± 66	215 ± 18
resPrP^D^ replicated	T1^20^ (To-2 -)	T1^21^ (To-2 -)	T1^21^ (To-2 +)	T2 (To-2 +)
**Tg129M** (1st pass.)				
Inoculum	VV1^20^	VV1^21^	VV1^21−20*^	VV2
Attack rate (%)	100	0	100	100
Incubation (dpi)	554 ± 53	0	570 ± 60	626 ± 56
resPrP^D^ replicated	T1^21−20^ (To-2 -)	No transmis	T1^21−20^ (To-2 +)	T1^20^ (To-2 -)
**Tg129M** (2nd pass.)				
Inoculum	VV1^20^	VV1^21^	VV1^21−20^*	ND
Attack rate (%)	100	0	100	ND
Incubation (dpi)	338 ± 30	0	292 ± 16	ND
resPrP^D^ replicated	T1^21−20^ (To-2: ND)	No transmis	T1^21−20^ (To-2: ND)	ND

resPrP^D^: proteinase K (PK)-resistant PrP^D^; dpi: days post-inoculation (mean value ± standard deviation). Tohoku-2 positive (To-2 +) or negative (To-2 −) immunoreactivity. VV1^21−20^*: sCJDVV1-2 with T2 accounting for ~ 5% of total resPrP^D^. Dpi (1st pass.), Tg129V: VV2 vs. VV1^20^, *P* < 0.0008; VV2 vs. VV1^21^, *P* < 0.04; VV1^20^ vs. VV1^21^ or VV1^21−20^*, *P* > 0.05. Dpi (1st pass.), Tg129M: VV2 vs. VV1^20^, *P* < 0.03; VV2 vs. VV1^21–20^* and VV1^20^ vs. VV1^21−20^*, *P* > 0.05; Dpi (2nd pass.), Tg129M: VV1^20^ vs. VV1^21−20^*, *P* < 0.009. Dpi, Tg129M: 1st vs. 2nd passage of VV1^20^ or VV1^21−20^*, *P* < 0.0001. Statistical significance was determined by one-way ANOVA and Student’s t-test. No transmis.: no transmission; pass.: passage; ND: not done

Tg129M mice showed a 100% attack rate following challenge with T1^20^, T1^21−20^* and T2, whereas T1^21^ failed to replicate. T1^20^ and T1^21−20^* essentially shared the incubation periods (554 ± 53 dpi and 570 ± 60 dpi, respectively), which, on average, were 1.7 times longer than those of Tg129V (Tables 1 and Additional file 5: S2). The incubation period following inoculation with T2 was nearly three times longer than Tg129V (Tables 1 and Additional file 5: S2). Overall, resPrP^D^ replication was much less accurate: T1^20^ replicated as T1^21−20^* with ~ 20 kDa preponderance and no detectable T2, whereas T1^21−20^* inoculation engendered both ~ 21 and ~ 20 kDa fragments but with an inverted ratio as compared to that of the inoculum, and with traces of T2 (Fig. 1b, d). Furthermore, T2 was replicated as T1^20^, which immunoreacted with the resPrP^D^ type non-specific 3F4 Ab but not with the resPrP^D^ T2-specific Tohoku-2 Ab, clearly indicating that the resPrP^D ^T2 of the inoculum was not replicated (Tables 1 and Additional file 5: S2, Fig. 1e). This finding contrasts with the apparently faithful replication of the  ~ 19 kDa T2 by the Tg129V mice inoculated with sCJDVV2, and it is puzzling considering that *bona fide* T2 ~ 19 kDa fragment is reproduced by Tg129M mice after inoculation with T1^21−20^* (Tables 1 and Additional file 5: S2, Fig. 1e). The second passage in Tg129M mice as the first, resulted in the replication of T1^20^ and T1^21−20^* only with indistinguishable electrophoretic profiles (Table 1 and Additional file 2: Figure S2). However, the incubation periods were respectively reduced ~ 1.6- and ~ 1.9-fold due to the strain adaptation (*P* < 0.0001). Furthermore, the ~ 50 days longer incubation period of mice inoculated with T1^20^ also was statistical significant (T1^20^: 338 ± 30 dpi; T1^21−20^*: 292 ± 16 dpi; *P* < 0.009) (Tables 1 and Additional file 5: S2).

### Histopathological and immunohistochemical features of inoculated Tg mice

**Fig. 2:**
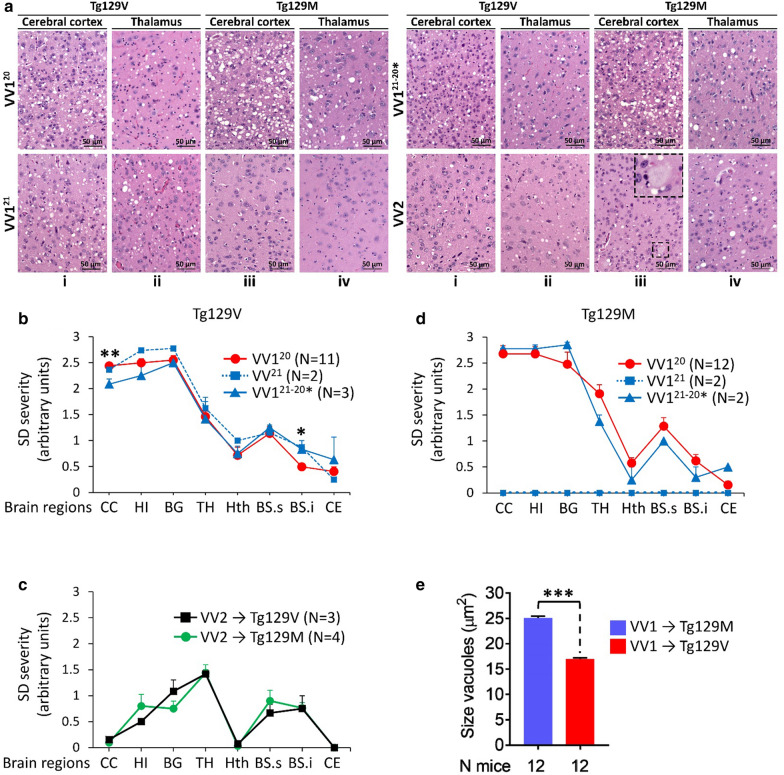
Histopathology, lesions profiles and vacuole size determinations. **a**: Hematoxylin and Eosin (H.E.) staining. VV1^20^, VV1^21^, VV1^21−20^* and VV2 refer to the inocula. **Tg129V, i**-**ii**, **VV1**^**20**^, **VV1**^**21**^ and **VV1**^21−20^*: Spongiform degeneration (SD) affecting more severely the cerebral cortex than thalamus. **i**-**ii**, **VV2**: Scant SD in the cerebral cortex and prominent in the thalamus. **Tg129M**, **iii**-**vi**, **VV1**^**20**^ and **VV1**^21−20^*: SD with large vacuoles. **iii**-**iv**, **VV1**^**21**^: Mouse brain free of lesions. **iii**-**iv**, **VV2**: Cortical plaques; inset, **iii**: high magnification of a plaque. **b** and **c**: Profiles of brain distribution and severity of SD were similar in Tg129V mice challenged with VV1^20^, VV1^21^, and VV1^21−20^* (**b**), and in Tg mice challenged with VV2 (**c**). **d**: Profiles in Tg129M mice inoculated with VV1^20^ and VV1^21−20^* were similar; VV1^21^-inoculated mice were free of lesions. **e**: Vacuole size averaged from nine VV1^20^ and three VV1^21−20^* challenged mice was ~ 8 µm^2^ greater in Tg129M than Tg129V. *P < 0.05, **P < 0.003, ***P < 0.0001. CC: Cerebral cortex, HI: hippocampus, BG: basal ganglia, TH: thalamus, Hth: hypothalamus, BS.s: brainstem, superior, BS.i: brainstem, inferior, CE: cerebellum

**Fig. 3:**
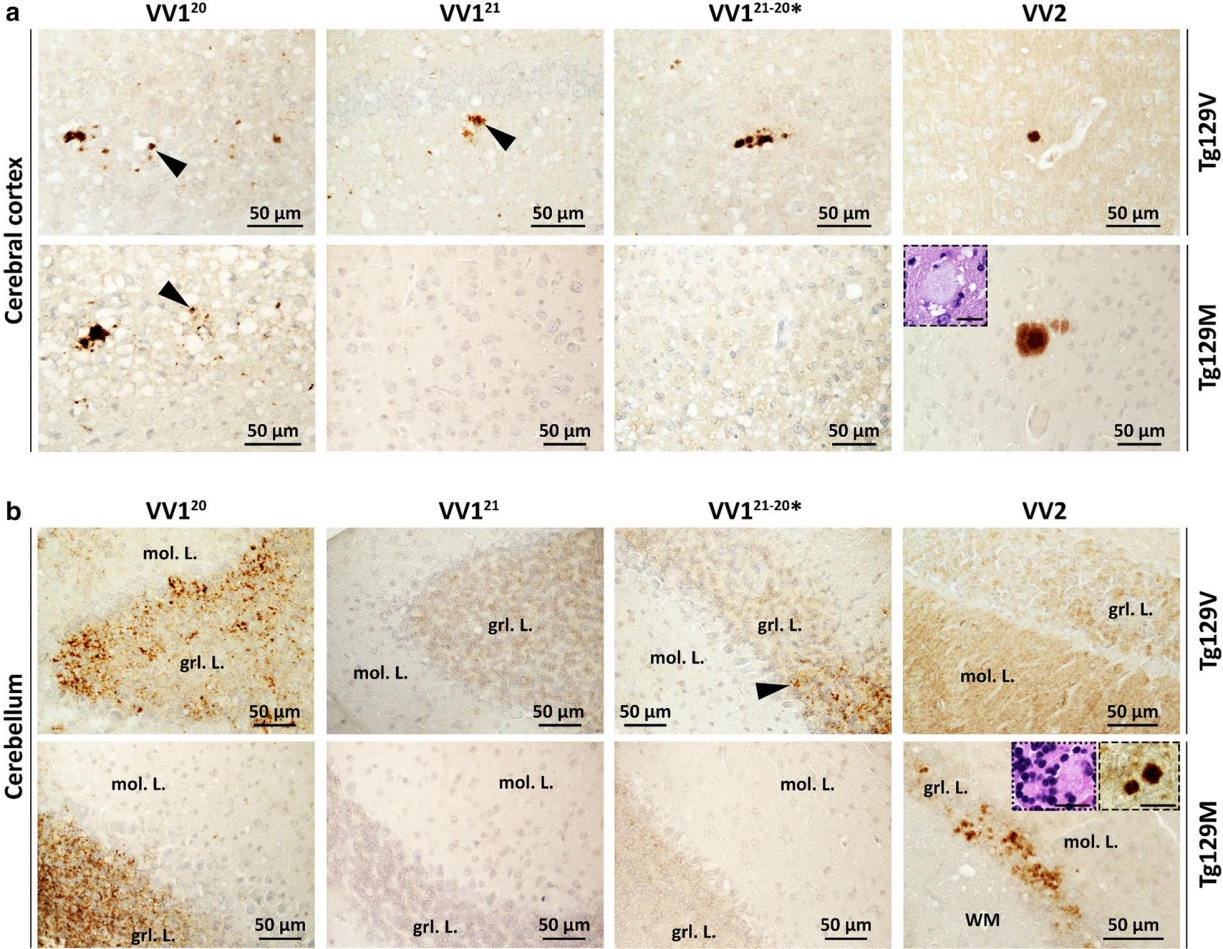
PrP immunohistochemistry (IHC). **a**: Cerebral cortex. **1st row, VV1**: PrP granular deposits (arrowheads) often distributed around the rim of vacuoles. **VV2**: A plaque-like PrP. **2nd row, VV1**^**20**^: PrP deposits co-distributing with SD; arrowhead: granular PrP. **VV1**^**21**^ and **VV1**^21−20^*: negative PrP IHC. **VV2**: A PrP plaque; inset: H.E. staining of the plaque. **b**: Cerebellum. **1st row, VV1**^**20**^ and **VV1**^21−20^*: PrP deposition affecting the granule cell layer (grl. L.); arrowhead, **VV1**^21−20^*: granular PrP. **VV1**^**21**^ and **VV2**: Negative PrP IHC; mol. L.: molecular layer. **2nd row, VV1**^**20**^: PrP deposition in grl. L. **VV1**^**21**^ and **VV1**^21−20^*: Negative PrP IHC. **VV2**: Plaque and plaque-like PrP ; dotted and dashed insets: two PrP plaques depicted on H.E. and IHC preparations, respectively. Scale bar insets: 20 µm; Ab: 3F4

Inoculations of resPrP^D^ T1^20^, T1^21^ and T1^21−20^* variants to Tg129V mice generated similar histopathological features (Table 2, Figs. 2, 3) consisting of prominent spongiform degeneration (SD) and astrogliosis of neocortex, hippocampus and basal ganglia, which progressively subsided caudally (except for a small peak in the brain stem) reaching the lowest level in the cerebellum. Vacuoles commonly were of medium or intermediate size, and plaques were not detected (Fig. 2a, b).

**Table 2:** Histopathological and PrP immunohistochemical (IHC) features of inoculated Tg mice (1st passage)

Mouse line	Inoculum	H.E			PrP IHC pattern	
		SD topography	Vacuole size	Plaques	Cerebral Cortex (CC)	Cerebellum
Tg129V	VV1^20^	↑CC- ↓Th^a^	Medium	No	Granul. Aggreg	Focal, Grl. L
	VV1^21−20^*	↑CC- ↓Th	Medium	No	Granul. Aggreg	Focal, Grl. L
	VV1^21^	↑CC- ↓Th	Medium	No	Granul. Aggreg	Negative
Tg129M	VV1^20^	↑CC- ↓Th	Large	No	Granul. Aggreg	Focal, Grl. L
	VV1^21−20^*	↑CC- ↓Th	Large	No	Negative	Negative
	VV1^21^	Negative				
Tg129V	VV2	↓CC- ↑Th	Small	Yes, BS	Plaque-like	Negative
Tg129M	VV2	↓CC- ↑Th	Small	Yes, widespread	Plaques	Plaques & plaque-like

^a^Arrows depict gradients of lesion severity: upward arrow = maximum; downward arrow = minimum; H.E.: Hematoxylin–eosin; SD: spongiform degeneration; CC: cerebral cortex; Th: thalamus; Granul. Aggreg.: granule aggregate; Grl. L.: granule cell layer; BS: brainstem

Matching PrP immunostaining (IHC) showed some topographic variation. In the cerebral cortex the pattern was similar in the three T1 variants and consisted of individual granules or clusters of variable sizes that co-distributed with SD (Table 2 and Fig. 3a). However, while in T1^21^ and T1^21−20^*, the granular deposits were limited to the cerebral neocortex and hippocampus, T1^20^ inoculated Tg mice displayed PrP granules also in subcortical regions. Furthermore, the cerebellum showed PrP deposition in the granule cell layer in T1^20^ and T1^21−20^* but it was entirely negative in T1^21^ Tg mice (Table 2, Fig. 3b).

In Tg129M mice inoculated with the T1^20^ or T1^21−20^* variants, SD severity and brain regional distribution or lesion profile, did not significantly differ from those of Tg129V mice (Fig. 2a, d, and Additional file 3: Figure S3 A and B) although vacuoles were significantly larger (*P* < 0.0001) (Fig. 2a, e). Mice challenged with T1^21^ were free of lesions up to ~ 700 dpi (Table 1 and Fig. 2). Lesion profiles and vacuole size in second passage Tg129M mice challenged with T1^20^ and T1^21−20^* overlapped with those of the first passage (data not shown).

In T1^20^ Tg129M, PrP IHC pattern with granular aggregates in the cerebral and cerebellar cortices mirrored that of matching Tg129V, while T1^21−20^* Tg mice showed rare granular aggregates in subcortical regions but not in the cerebral cortex and cerebellum (Fig. 3a, b). No plaques were detected (Table 2 and Fig. 3b).

Following T2 inoculation, both Tg129V and Tg129M mice showed scant SD that, contrary to T1 variants, displayed an inversed severity gradient that increased progressively from the cerebral cortex, where it was virtually absent, to the thalamus (Table 2, Fig. 2a, c). Furthermore, SD was made of small vacuoles. In the cerebellum, astrogliosis also was significantly more severe than that observed in mice inoculated with T1 variants (Additional file 3: Figure S3 C and E) although granule cell depopulation did not reach statistical significance (Additional file 3: Figure S3D and E). PrP IHC showed plaques-like aggregates in the cerebral cortex of the Tg129V mice while real plaques were seen only in the brain stem and septal nuclei in one mouse (Figs. 3 and Additional file 4: S4). By contrast, plaques were widespread in Tg129M mice and populated the cerebral cortex, thalamus, the border between the hippocampal alveus and the corpus callosum, the brain stem and cerebellum in the majority (70%) of the inoculated mice (Figs. 3 and Additional file 4: S4).

## Discussion

Previous transmission studies to Tg mice expressing human wild-type or mutated PrP did not examine the mouse replications of the sCJDVV1 T1 variants that we have recently described [4, 12, 13, 16, 21, 24, 26, 34, 45]. We now show that T1^20^ and T1^21^ are faithfully reproduced in Tg129V mice with no significantly different incubation periods and slightly different histotypes reminiscent of that associated with the -VV1 subtype (Tables 1, 2). Transmissibility characteristics clearly distinguished T1^20^ from T1^21^ following inoculation to the Tg129M mice where T1^20^ accumulated as T1^21−20^* whereas T1^21^ was not detected.

T1^20^ and T1^21−20^* transmission to Tg129M required an incubation period nearly 60% longer than that of the Tg129V mice consistent with the effect of the 129 genotype barrier. This assumption is further supported by the significant reduction in the incubation period following second passage in Tg129M with T1^20^ and T1^21−20^* (Tables 1 and Additional file 5: S2). A similar phenomenon has been observed following second passage of sCJDVV1 prions to Tg129M mice [12].

In contrast to the accurate reproduction of the T1^20^ and T1^21^ variants, T1^21−20^* inoculated to Tg129V mice accumulated as T1^21^. Conversely, Tg129M mice faithfully accumulated T1^21−20^*; both mouse lines accumulated T1^21−20^* with the additional presence of T2 traces (also present in the inoculum) which may have impacted the replication. The two T1^21−20^* variants generated in Tg129M following inoculation of T1^20^ and T1^21−20^*, respectively, had significantly different incubation period on second passage and differed in the histotype based on the lack of cerebral cortical and cerebellar pathology in the latter. Furthermore, second passage in Tg129M mice confirmed the lack of transmission of T1^21^. An unexpected phenotypical distinction between T1 inoculated Tg129V and− 129M mice was the size of the vacuoles, which was significantly larger in the Tg129M mice consistent with an effect of the PrP 129MV polymorphism on this distinctive histopathological feature. Vacuole size and lesion profiles were virtually identically in Tg129M mice of the 1st and 2nd passage.

Transmission of sCJDVV2 T2 used as control revealed expected results. In contrast to the faithful replication of -VV2 T2 by the Tg129V mice, a T1^20^ variant was reproduced in the Tg129M after an incubation period that was three times longer than that in Tg129V. Our data resemble those recently described in a transmission study employing the same Tg129M mouse line as in our study [12]. These findings confirm the incompetence of human PrP^C^-129M to reproduce -VV2 T2 [12, 24] as opposed to the faithful transmission of -MM2 to Tg129M mice [30, 34].

The original classification of major sCJD subtypes based on histotype and PrP^D^ characteristics has undergone recent revisions [2, 11, 12, 27, 32, 43]. Sporadic CJDMM(MV)1 (a combination of -MM1 and -MV1, which share histotype and PrP^D^ characteristics) as well as -MM2 (also referred to as MM2C) and -VV2, are seen as definitely distinct subtypes [5, 18, 19, 32]. They are associated with PrP^D^ variants that show distinct conformational and transmissible characteristics but have straightforward electrophoretic profiles of either PrP^D^ type 1 or 2. By contrast, the -MV2 and -VV1 subtypes have shown considerable electrophoretic heterogeneity [11, 32, 33]. The subtype -MV2 is now subdivided into two variants; the first, -MV2C, is currently viewed as a phenocopy of -MM2 in terms of histotype and PrP^D^ characteristics; the second, -MV2K, is characterized by the presence of kuru (K) plaques and heterogeneous PrP^D^ inclusive of at least two components: (i) a ~ 19 kDa PrP^D^ variant with gel mobility and conformational features similar to the -VV2 ~ 19 kDa, and (ii) a ~ 20 kDa PrP^D^ (also termed “intermediate” type or “type i”) of uncertain origin. Recently, however, the convergence of transmission and mass spectrometry data basically indicates that (i) the -MV2C and -MV2K phenotypes and respective PrP^D^ characteristics are directly related to the representation of the resPrP^D^-129M and -129V components, respectively [32], (ii) the -MV2K ~ 20 kDa variant is made exclusively of the minority resPrP^D^-129 M component, and (iii) the -MV2K ~ 20 kDa appears to be an adaptation of the VV2 PrP^D^ type 2 to the 129MM or 129MV background ([24, 25, 32] and this study).

Our previous study showed that in sCJDVV1 resPrP^D^ presents an even higher level of complexity given that it features three combinations of resPrP^D^ kDa: T1^20^, T1^21^ and T1^21−20^ [11]. The T1^20^ and T1^21^ difference of ~ 1 kDa in electrophoretic mobility of the two resPrP^D^ variants, although minor, is not negligible since it implies that the span of the PK-resistant region (i.e., the abnormal secondary structure generated during the PrP^C^ to PrP^D^ conversion) is different in the T1^20^ and T1^21^ variants. Indeed, T1^20^ and T1^21^ isolated from sCJDVV1 brains show features (e.g., resistance to enzymatic degradation by PK and propensity to unfold following exposure to the denaturing agent guanidine hydrochloride) that differ significantly, which further supports the conclusion that these two T1 variants have distinct conformational characteristics even though they are associated with similar histotypes [11]. It is noteworthy that the association of conformationally distinct prions strains with similar phenotypes has been previously reported [1, 44]. Our present findings are consistent with this conclusion given that both T1^20^ and T1^21^ can be faithfully replicated in Tg129V mice but display opposite transmission characteristic in Tg129M mice; furthermore, mimicking sCJDVV1, T1^20^ and T1^21^ are associated with essentially similar histotypes in the Tg mice.

Our study also offers the opportunity to directly compare the histotype of the T1^20^ variant associated with -VV1 with that of the T1^20^ variant generated after inoculation of -MV2K and -VV2 PrP^D^ to Tg129M mice [24, 25]. Tg129M mice inoculated with T1^20^ from -VV1 subjects are characterized by medium size vacuole SD, predominantly impacting the cerebral cortex, and lack of PrP plaques. By contrast, Tg129M inoculated with -MV2K and -VV2 isolates (also reported to harbor a T1^20^ variant) [24] displayed ubiquitous plaques along with small vacuole SD occupying mostly subcortical regions. These two distinct histotypes are thus reminiscent of the -VV1 and -MV2K/-VV2 subtypes, respectively. The nature of the molecular features—besides the M and V incongruity at PrP residue 129—underpinning the complex and major impact on the histotype associated with the two T1^20^ variants, remains to be resolved.

## Conclusions

The present study further contributes to understand the molecular features of T1 variants in sCJDVV1 [11]. Our present data along with the previous conformational studies are consistent with the conclusion that T1^21^ and T1^20^ resPrP^D^ are two distinct human prion strains that generate similar clinico-histopathological phenotypes. The lack of transmissibility of T1^21^ VV1 to Tg129M mice suggests that subjects with the PrP-129MM genotype may not be at risk of acquiring prion disease from sCJDVV1 donor harboring the T1^21^ variant. Understanding the molecular properties of PrP^D^ T1 associated with sCJDVV1 may shed light into the common early presentation of this subtype and be essential for strain-sensitive therapeutic approaches [3, 15, 20, 29].

## Competing interests

The authors declare that they have no competing interest.

## Supplementary Information

**Additional file 1. Fig. S1**: Western blot profile of resPrP^D^ from sCJDVV cases used as inocula. **A**: Immunoblot with 3F4 antibody. Lane 1–3: One of the three sCJDVV1 inocula with T1 unglycosylated (unglyc.) isoform migrating to ~ 20 kDa (VV1^20^, lane 1), and sCJDVV2 with T2 unglyc. resPrP^D^ of ~ 19 kDa (VV2, lanes 2, 3). Lane 4: sCJDVV1 with T1 resPrP^D^ migrating to ~21 kDa (VV1^21^). Lane 5: sCJDVV1-2 control with co-existing T1^21^ and T2 resPrPD fragments. Lane 6: sCJDVV1-2 inoculum (VV1^21–20*^) harboring a ~ 21–20 kDa doublet with prominent ~21 kDa band; T2 is not detected by 3F4. Lane 7: sCJDVV2 control. **B**: Immunoblot with 1E4 antibody. Lanes 1-4: 1E4 immunoreacted with T1 populating VV1^20^ (lane 1) and VV1^21^ (lane 3) inocula, and T2 harvested from VV2 (lanes 2 & 4). Lane 5: 1E4 detected a faint band of ~19 kDa in addition to the ~21-20 kDa doublet in VV1^21–20*^. Put.: putamen; CC: cerebral cortex.

**Additional file 2. Fig. S2:** Characterization of mouse brain resPrP^D^ following 2nd passage in Tg129M mice. T1 and its superscript atop the blot refer to the mouse resPrP^D^ T1 variant; VV1^20^ and VV1^21^ refer to resPrP^D^ harvested from sCJDVV1 controls. Mouse resPrP^D^ showing a ~21–20 kDa doublet following 2nd passage with VV1^20^ (lanes 2 & 3) and VV1^21–20*^ (lanes 5 & 6); lane 6: longer exposure time of resPrP^D^ visualized in lane 5. No resPrP^D^ was detected after serial passage with VV1^21^ (lane 7); Neg.: negative. Licor near-infrared (lanes 1-6); chemiluminescence (lanes 7 & 8).

**Additional file 3 Fig. S3:** Lesions profiles and assessment of cerebellar pathological changes. **A** and **B**: Tg129V and Tg129M mice challenged with sCJD VV1^20^ (**A**) or VV1^21–20*^ (**B**) generated similar lesion profiles. **C** and **D**: Severity scores of gliosis (**C**) and neuronal loss (**D**) in the granule cell layer of the cerebellum in mice challenged with sCJD VV1^20^, VV1^21^, VV1^21–20*^ (averaged values) and VV2. **E**: Representative microphotographs showing gliosis and loss of granule cells in the cerebellum of Tg129V mice challenged with VV1^20^ and VV2, respectively; arrows: astrocytes; *P<0.05. **P<0.02. Each point of the profile in **A** and **B**, and bar graphs in **C** and **D** are expressed as mean ± SEM.

**Additional file 4 Fig. S4:** Histopathology and PrP immunohistochemistry (IHC) in mice inoculated with sCJDVV2. **i** and **iii**: H.E. staining; **ii** and **iv**: PrP IHC. 1st row, **i** and **ii**: The cerebral cortex (CC), alveus (alv) and hippocampal CA1 regions were free of plaques and generated a negative PrP immunostaining. **iii** and **iv**: An aggregate (arrow) visible at H.E. (**iii**) was positively stained by an antibody (Ab) to PrP (**iv**). 2nd row, **i** and **ii**: Aggregates of plaques (**i**) affecting the lower brainstem immunoreacted with an Ab to PrP (**ii**); inset, **i**: higher magnification of congregate plaques. **iii** and **iv**: Plaques (arrowheads) distributed in a diagonal row in the upper brainstem; inset, **iii** and **iv**: a rounded plaque. Scale bar insets: 100 µm (1st row, **iv**) and 20 µm (2nd row, **i**); Ab: 3F4.

Additional file5 (DOCX 39 KB)

## Data Availability

Data used in this study are available from the corresponding author on reasonable request.

## Abbreviations

CJD: Creutzfeldt–Jakob disease
sCJD: Sporadic CJD
T1: Type 1
T2: Type 2
PrP: Prion protein
PrP^D^: Disease-associated PrP
PK: Proteinase K
resPrP^D^: PK-resistant PrP^D^
T1^21^ and T1^20^: T1 variants with unglycosylated resPrP^D^ isoform of ~ 21 and ~ 20 kDa, respectively
T1^21^^−^^20^: T1 variant with unglycosylated resPrP^D^ doublet of~ 21–20 kDa
VV1^21^, VV1^20^ and VV1^21^^−^^20^*: Shown in all figures and Tables 1 and 2 refer to the human sCJDVV cases
M: Methionine
V: Valine
Tg129V and Tg129M: Transgenic mice expressing PrP-129V and PrP-129M, respectively
Ab: Antibody
BH: Brain homogenate
H.E.: Hematoxylin–eosin staining
PrP IHC: PrP immunostaining or immunohistochemistry
SD: Spongiform degeneration
WB: Western blot
dpi: Days post-inoculation
