# Supplementary figures and images for: Two distinct conformers of PrPD type 1 of sporadic Creutzfeldt–Jakob disease with codon 129VV genotype faithfully propagate in vivo
Source: Acta Neuropathol Commun. 2021 Mar 25;9:55. doi: 10.1186/s40478-021-01132-7 (PMC7995586; doi:10.1186/s40478-021-01132-7)

A

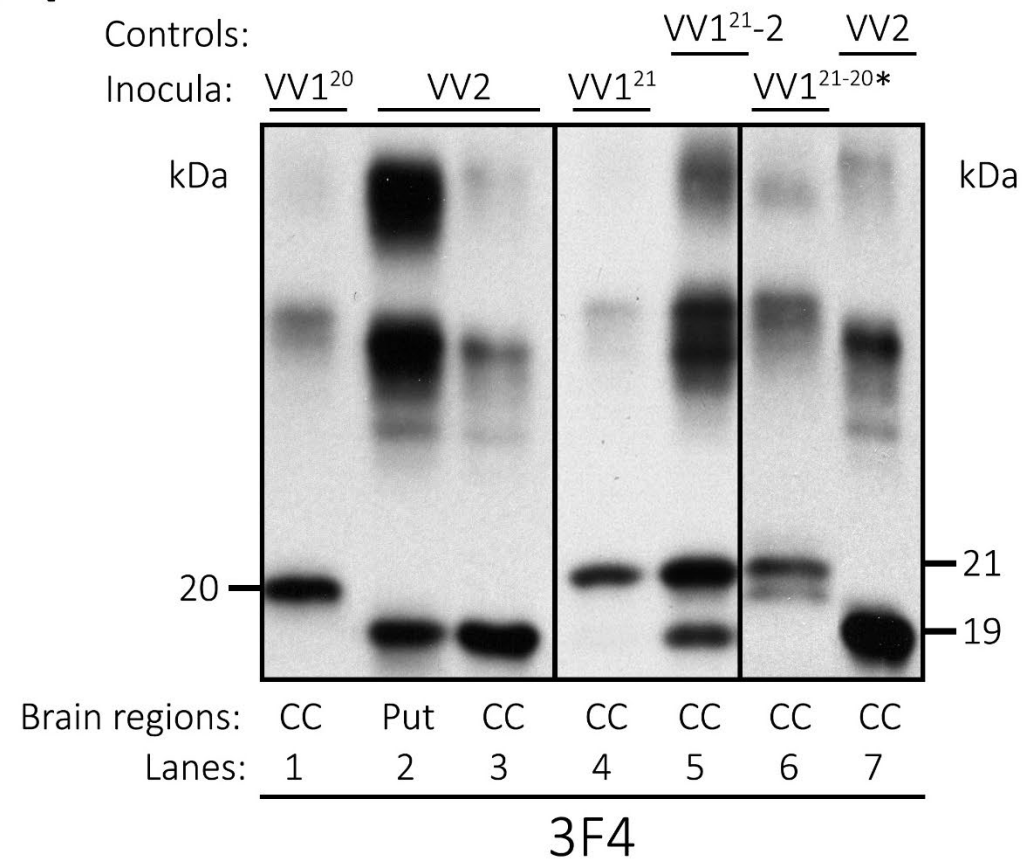

B

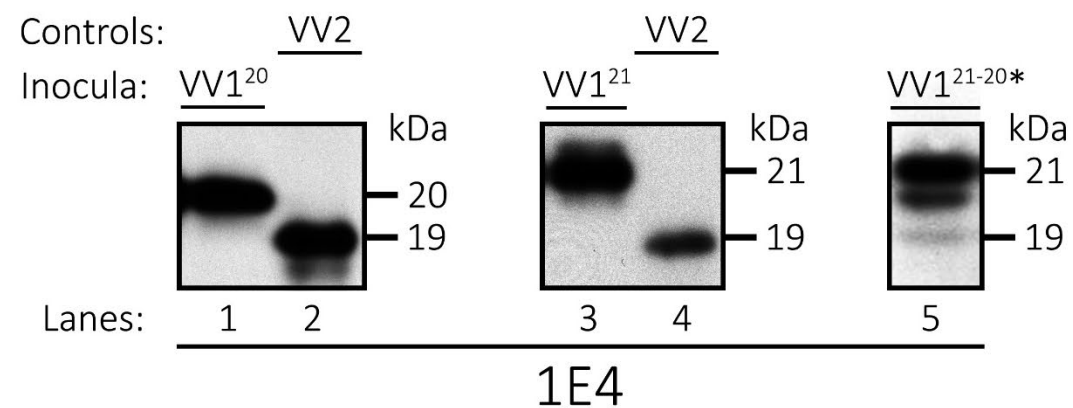

Supplement: Supplementary file 1 — Additional file 1. Fig. S1: Western blot profile of resPrPD from sCJDVV cases used as inocula. A: Immunoblot with 3F4 antibody. Lane 1–3: One of the three sCJDVV1 inocula with T1 unglycosylated (unglyc.) isoform migrating to ~ 20 kDa (VV120, lane 1), and sCJDVV2 with T2 unglyc. resPrPD of ~ 19 kDa (VV2, lanes 2, 3). Lane 4: sCJDVV1 with T1 resPrPD migrating to ~21 kDa (VV121). Lane 5: sCJDVV1-2 control with co-existing T121 and T2 resPrPD fragments. Lane 6: sCJDVV1-2 inoculum (VV121–20*) harboring a ~ 21–20 kDa doublet with prominent ~21 kDa band; T2 is not detected by 3F4. Lane 7: sCJDVV2 control. B: Immunoblot with 1E4 antibody. Lanes 1-4: 1E4 immunoreacted with T1 populating VV120 (lane 1) and VV121 (lane 3) inocula, and T2 harvested from VV2 (lanes 2 & 4). Lane 5: 1E4 detected a faint band of ~19 kDa in addition to the ~21-20 kDa doublet in VV121–20*. Put.: putamen; CC: cerebral cortex. [file 40478_2021_1132_MOESM1_ESM.pdf]

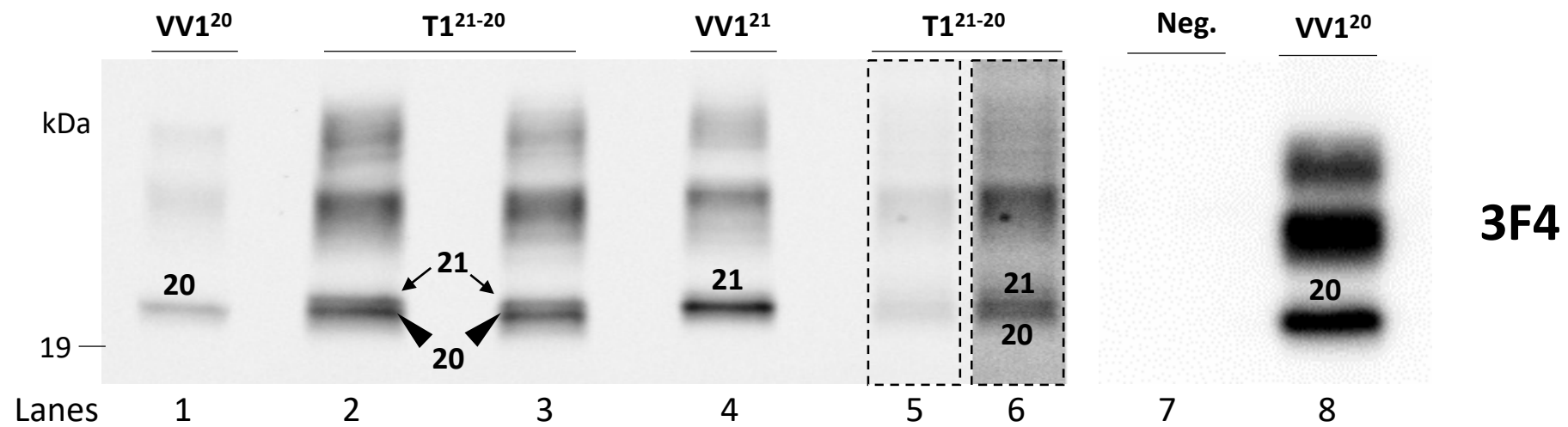

Supplement: Supplementary file 2 — Additional file 2. Fig. S2: Characterization of mouse brain resPrPD following 2nd passage in Tg129M mice. T1 and its superscript atop the blot refer to the mouse resPrPD T1 variant; VV120 and VV121 refer to resPrPD harvested from sCJDVV1 controls. Mouse resPrPD showing a ~21–20 kDa doublet following 2nd passage with VV120 (lanes 2 & 3) and VV121–20* (lanes 5 & 6); lane 6: longer exposure time of resPrPD visualized in lane 5. No resPrPD was detected after serial passage with VV121 (lane 7); Neg.: negative. Licor near-infrared (lanes 1-6); chemiluminescence (lanes 7 & 8). [file 40478_2021_1132_MOESM2_ESM.pdf]

**A**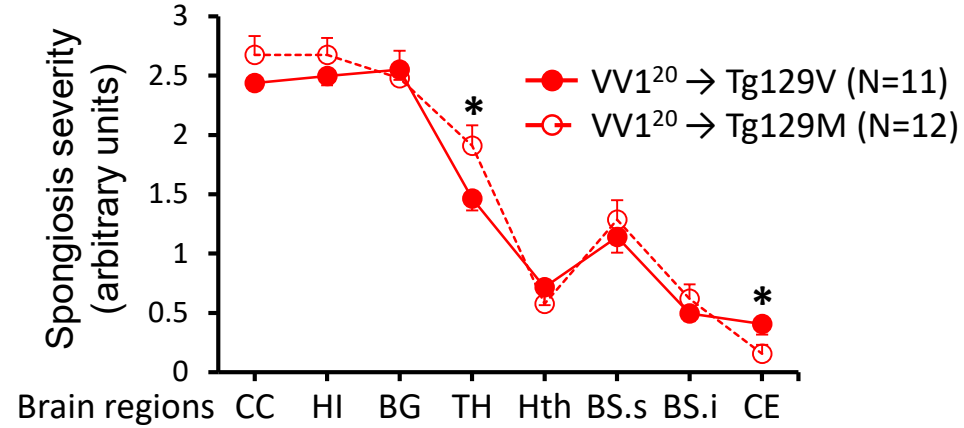**B**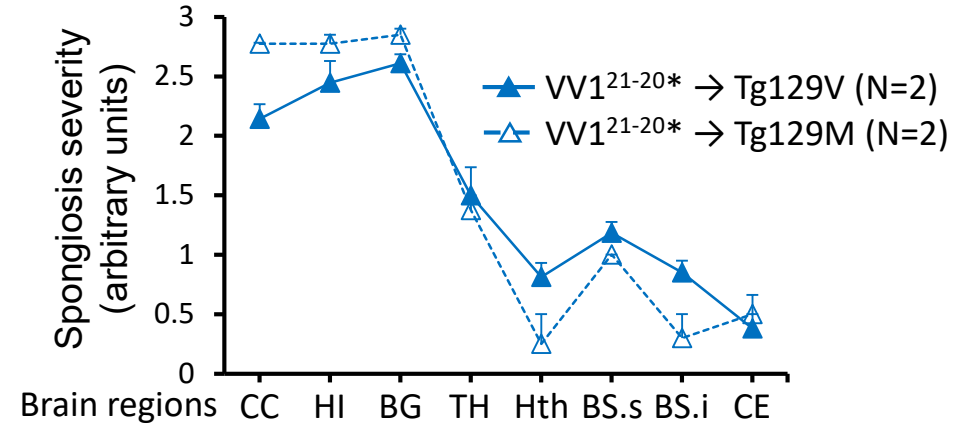**C**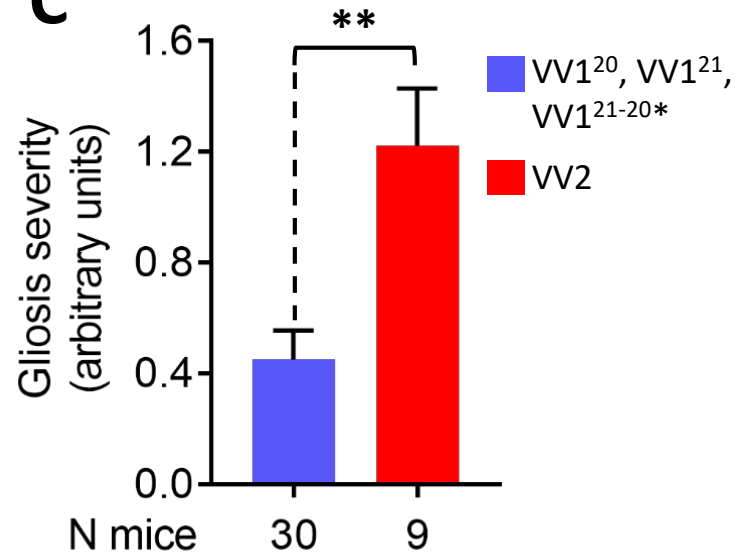**D**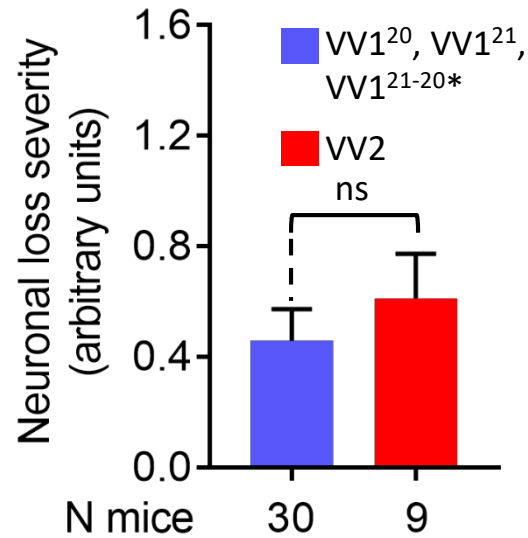**E**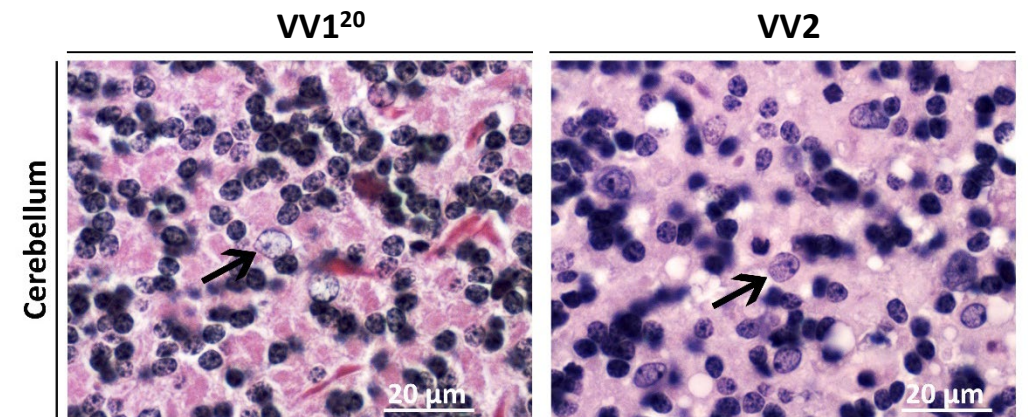

Supplement: Supplementary file 3 — Additional file 3 Fig. S3: Lesions profiles and assessment of cerebellar pathological changes. A and B: Tg129V and Tg129M mice challenged with sCJD VV120 (A) or VV121–20* (B) generated similar lesion profiles. C and D: Severity scores of gliosis (C) and neuronal loss (D) in the granule cell layer of the cerebellum in mice challenged with sCJD VV120, VV121, VV121–20* (averaged values) and VV2. E: Representative microphotographs showing gliosis and loss of granule cells in the cerebellum of Tg129V mice challenged with VV120 and VV2, respectively; arrows: astrocytes; *P<0.05. **P<0.02. Each point of the profile in A and B, and bar graphs in C and D are expressed as mean ± SEM. [file 40478_2021_1132_MOESM3_ESM.pdf]

## VV2

Tg129V

Tg129M

Cerebral cortex

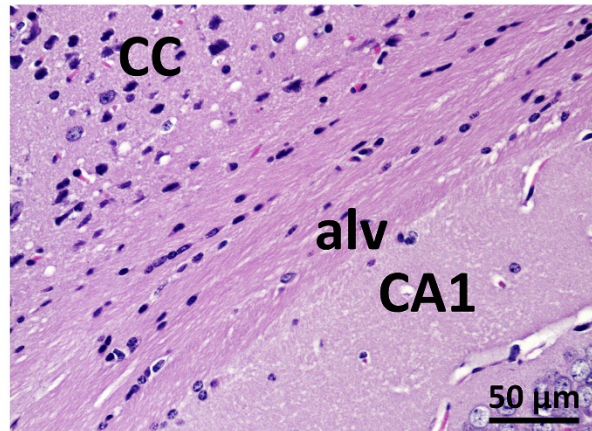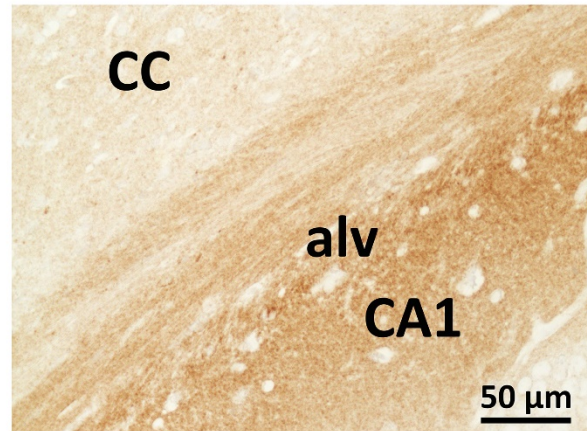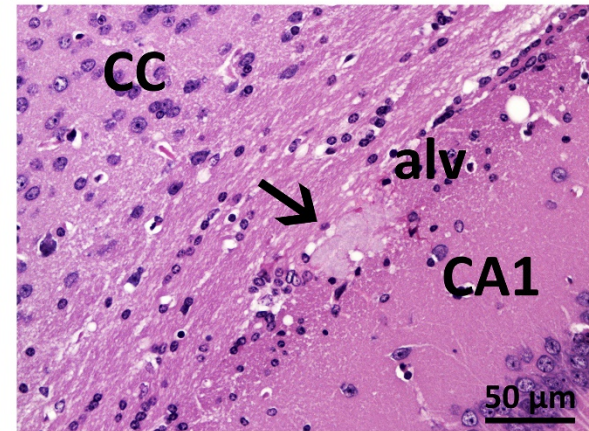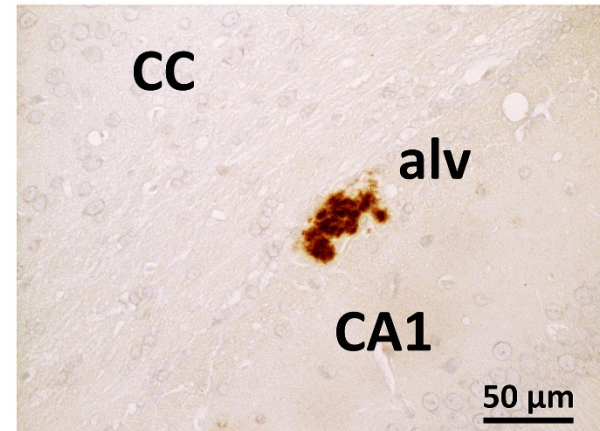

Brainstem

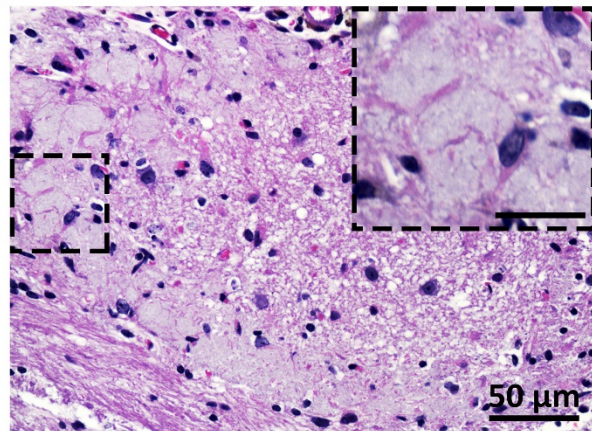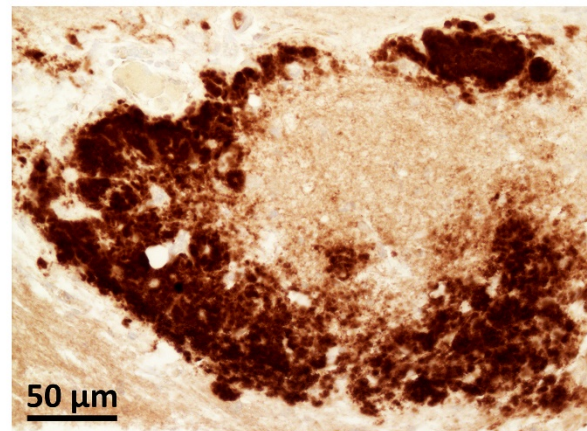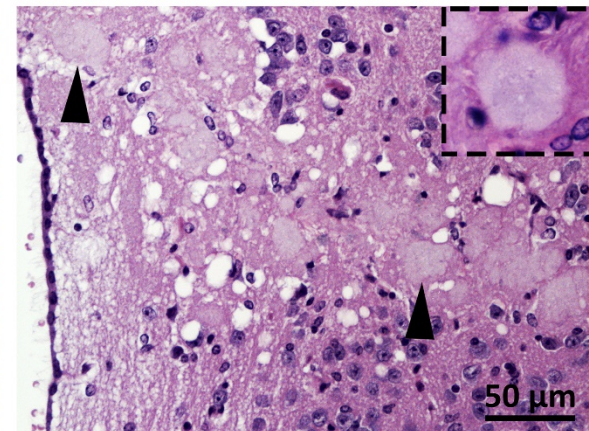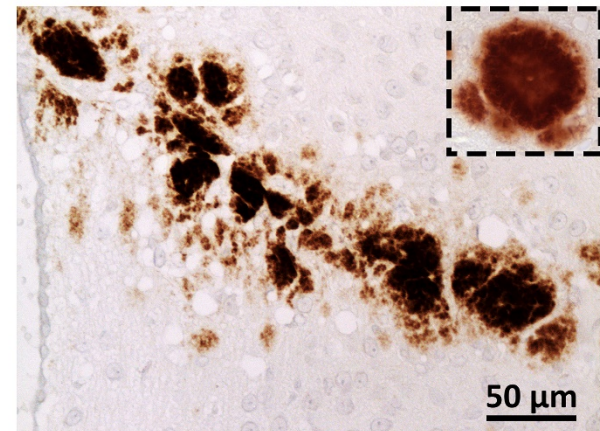

i

ii

iii

iv

Supplement: Supplementary file 4 — Additional file 4 Fig. S4: Histopathology and PrP immunohistochemistry (IHC) in mice inoculated with sCJDVV2. i and iii: H.E. staining; ii and iv: PrP IHC. 1st row, i and ii: The cerebral cortex (CC), alveus (alv) and hippocampal CA1 regions were free of plaques and generated a negative PrP immunostaining. iii and iv: An aggregate (arrow) visible at H.E. (iii) was positively stained by an antibody (Ab) to PrP (iv). 2nd row, i and ii: Aggregates of plaques (i) affecting the lower brainstem immunoreacted with an Ab to PrP (ii); inset, i: higher magnification of congregate plaques. iii and iv: Plaques (arrowheads) distributed in a diagonal row in the upper brainstem; inset, iii and iv: a rounded plaque. Scale bar insets: 100 µm (1st row, iv) and 20 µm (2nd row, i); Ab: 3F4. [file 40478_2021_1132_MOESM4_ESM.pdf]
