# Supplementary material for: Two distinct conformers of PrPD type 1 of sporadic Creutzfeldt–Jakob disease with codon 129VV genotype faithfully propagate in vivo
Source: Acta Neuropathol Commun. 2021 Mar 25;9:55. doi: 10.1186/s40478-021-01132-7 (PMC7995586; doi:10.1186/s40478-021-01132-7)
Supplement: Supplementary file 5 — Additional file5 (DOCX 39 KB) [file 40478_2021_1132_MOESM5_ESM.docx]

**Supplementary materials**

**Table S1**. Demographics and resPrP^D^molecular features of sCJDVV cases used as inocula

| **sCJD**  **subtype** | **Age at onset**  **(years)** | | **Disease duration (months)** | | **Inoculum** | | | | |
| --- | --- | --- | --- | --- | --- | --- | --- | --- | --- |
|  |  |  |  |  | **Brain region** | | **resPrP^D^ T1**  **variant** | | **Amount of resPrP^D^ T2 (1E4 Ab)** |
| **VV1** | |  | |  | |  | |  |  |
| Case 1 | 25 | | 8 | | frontal cortex | | T1^20^ | | 0 |
| Case 2 | 30 | | 10 | | parietal cortex | | T1^20^ | | 0 |
| Case 3 | 34 | | 13 | | frontal cortex | | T1^20^ | | 0 |
| Case 4 | 38 | | 12 | | frontal cortex | | T1^21^ | | 0 |
| **VV1-2** | |  | |  | |  | |  |  |
| Case 5 | 71 | | 18 | | occipital cortex | | ^a^T1^21-20^* | | 5 |
| **VV2** | |  | |  | |  | |  |  |
| Case 6 | 64 | | 8 | | frontal cortex | | T2 | | 100 |
|  |  |  |  |  | putamen | |  |  |  |

^a^ Refers to resPrP^D^ T1 with a ~21-20 kDa doublet and prominent ~21 kDa fragment.

**Table S2**. Inoculation of sCJDVV prions to Tg129V and Tg129M mice

| **Inoculum** | |  |  | **H.E./IHC** |  |  |  | **Western blot** | |  |
| --- | --- | --- | --- | --- | --- | --- | --- | --- | --- | --- |
| **sCJD**  **subtype** | **resPrP^D^**  **type (T)^a^** | **Tg(HuPrP)**  **genotype**  **codon 129** | **No. with**  **clinical signs/total** | **No. positive/**  **total** |  | **dpi** | **No. positive/**  **total** | **resPrP^D^ size (kDa)** | | |
|  |  |  |  |  |  |  |  | **3F4** | **Tohoku-2** | |
| VV1 (1^st^ pass.) | T1^20^ | VV | 4/4 | 3/3 |  | 362±18^b^ | 4/4 | 20 | Neg. | |
|  | T1^20^ |  | 6/6 | 5/5 |  | 344±15 | 6/6 | 20 | Neg. | |
|  | T1^20^ |  | 7/7 | 3/3 |  | 351±13 | 7/7 | 20 | Neg. | |
|  | T1^21^ |  | 4/4 | 2/2 |  | 425±66 | 4/4 | 21 | Neg. | |
| VV1-2 (1^st^ pass.) | T1^21-20^* | VV | 4/4 | 4/4 |  | 315±66 | 4/4 | 21 | 19 | |
| VV2 (1^st^ pass.) | T2^c^ | VV | 4/4 | 4/4 |  | 215±18 | 4/4 | 19 | 19 | |
|  | T2^d^ |  | 5/5 | 3/3 |  | 214±15 | 5/5 | 19 | 19 | |
| VV1 (1^st^ pass.)  (2^nd^ pass.) | T1^20^ | MM | 5/5 | 3/3 |  | 547±64 | 5/5 | 21-20^e^ | Neg. | |
|  | T1^20^ |  | 5/5 | 4/4 |  | 569±64 | 5/5 | 21-20^e^ | Neg. | |
|  | T1^20^ |  | 6/6 | 5/5 |  | 547±41 | 6/6 | 21-20^e^ | Neg. | |
|  | T1^21^  T1^20^  T1^20^  T1^20^  T1^21^ |  | 0/6  7/7  6/6  7/7  0/5 | 0/2  7/7^g^  6/6^g^  7/7^g^  0/5^g^ |  | >650^f^  333±26  341±30  340±39  >650 | 0/6  7/7  6/6  6/6  0/5 | Neg.  21-20^e^  21-20^e^  21-20^e^  Neg. | Neg.  ND  ND  ND  ND | |
| VV1-2 (1^st^ pass.)  (2^nd^ pass.) | T1^21-20^* | MM | 4/4  4/4 | 2/3  4/4^g^ |  | 570±60  292±16 | 4/4  4/4 | 21-20^e^  21-20^e^ | 19  ND | |
| VV2 (1^st^ pass.) | T2 | MM | 6/6 | 3/3 |  | 626±56 | 6/6 | 20 | Neg. | |

^a^ Refers to resPrP^D^ type 1 (T1) with unglycosylated isoform migrating to i) ~20 kDa (T1^20^), ii) ~21 kDa (T1^21^), iii) ~21-20 kDa (T1^21-20^ with predominant ~21 kDa band) and co-existing type 2 (T2). ^b^ Expressed as mean ± standard deviation; ^c,d^ T2 harvested from ^c^ frontal cortex and ^d^ putamen of the same sCJDVV2 case. ^e^ Refers to T1^21-20^ with ~20 kDa band preponderance. ^f^ One mouse was found dead at 340 days post-inoculation (dpi). ^g^ Only H.E. available. Age-matched, uninoculated mice carrying the PrP-129M (N=2) and PrP-129V (N=2) genotypes were all negative. H.E.: Hematoxylin-eosin; IHC: PrP immunohistochemistry; No.: number; Neg.: negative; pass.: passage; ND: not done.
